# Advances in molecular tools for elucidating nucleic acid biology in fungal pathogens

**DOI:** 10.1093/femsml/uqag008

**Published:** 2026-03-12

**Authors:** Lukas Schrettenbrunner, Neshima Mavani, Slavica Janevska, Matthew G Blango

**Affiliations:** Junior Research Group RNA Biology of Fungal Infections, Leibniz Institute for Natural Product Research and Infection Biology—Hans Knöll Institute (Leibniz-HKI), 07745 Jena, Germany; Junior Research Group (Epi-)Genetic Regulation of Fungal Virulence, Leibniz Institute for Natural Product Research and Infection Biology—Hans Knöll Institute (Leibniz-HKI), 07745 Jena, Germany; Junior Research Group (Epi-)Genetic Regulation of Fungal Virulence, Leibniz Institute for Natural Product Research and Infection Biology—Hans Knöll Institute (Leibniz-HKI), 07745 Jena, Germany; Junior Research Group RNA Biology of Fungal Infections, Leibniz Institute for Natural Product Research and Infection Biology—Hans Knöll Institute (Leibniz-HKI), 07745 Jena, Germany

**Keywords:** DNA, RNA, chromatin, mycology, fungal infection, CRISPR

## Abstract

Throughout the history of molecular biology, surprising advances have come from the study of all sorts of microbes. The first description of DNA polymerase came from the bacterial workhorse *Escherichia coli*, reverse transcriptase was revealed by studies of the Rous Sarcoma Virus, and even the initial discovery of DNA as the hereditary molecule was determined using strains of pneumococci in the classic Griffith and Avery–MacLeod–McCarty experiments. Here, we build from these foundational discoveries to discuss the rapid development of molecular tools to study microbes themselves, with a focus on nucleic acid biology. We use fungal pathogens as a case study, as their diversity, complexity, and emerging appreciation as a global threat to society makes them particularly compelling and informative. In this review, we will address how advancements in methods to probe nucleic acids are now informing our understanding of fungal pathogens and the way we fight them in both the clinic and agriculture. We begin with DNA, taking a close look at the exciting progress in the fields of genetic engineering and chromatin biology, and their impact on the elucidation of virulence-associated cellular processes. Emerging RNA-based technologies follow, highlighting the value provided by biochemical advances and large-scale -omics approaches. We end by speculating on the future of molecular mycology and how these new approaches may facilitate generation of novel antifungals and diagnostic strategies.

## Teaser

Check out this new review highlighting advances in DNA-, RNA-, and chromatin-based technologies and how they are used to keep pace with the emerging threat of fungal pathogens.

## Introduction

The kingdom Fungi harbors the colloquially known yeasts, molds, and mushrooms; organisms that range from microscopic to macroscopic and greatly impact human society. Fungal species, likely numbering in the millions (Hawksworth and Lucking 2017), are important contributors to everything from environmental carbon cycling to food production in the form of, e.g. bread, beer, cheese, and of course mushrooms (Case et al. 2022). Mycorrhizal fungi support plant growth and subsequently human agriculture, while other fungi are used in bioremediation or as raw building materials (Niego et al. 2023). These examples only begin to demonstrate the breadth of influence of this diverse kingdom.

Fungi also pose a dangerous threat to our health, ecosystems, and food productive capacity, e.g. causing deadly infections in immunocompromised/-suppressed individuals and reducing crop yields (Fisher et al. 2020, Case et al. 2022, Guerreiro and Stukenbrock 2025). Despite the millions of fungi, only several hundred are thought to be pathogenic. Fungal infections of plants, livestock, and insect populations pose significant societal and economic dangers (Fisher et al. 2020). As a few examples, *Botrytis cinerea, Batrachochytrium* spp., and *Beauveria bassiana* are widespread, dangerous pathogens of plants, amphibians, and insects, respectively, with *Batrachochytrium* even thought to contribute to extinction events in amphibian populations (Cheng 2011). Fusaria are notorious pathogens of essentially every staple crop, threatening food security. Quite prominently, *Fusarium oxysporum* f. sp. *cubense* tropical race (TR) 4 (reclassified as *F. odoratissimum*) recently broke the resistance of Cavendish, the banana cultivar currently covering the global demand for this staple food (Ploetz 2015).

The World Health Organization published a Fungal Priority Pathogens list in 2022 to bring attention to the dangers of fungi to human health (World Health Organization 2022, Fisher and Denning 2023). Critical priority pathogens *Aspergillus fumigatus, Candida albicans, Candida auris* (syn. *Candidozyma auris*), and *Cryptococcus neoformans* were deemed most dangerous, due to high case numbers and rates of mortality, breadth of distribution, and increasing incidence of resistance, among other factors (Denning 2024). Additional less prevalent pathogens made up the high and medium groups, featuring, e.g. pathogens from the *Mucorales, Fusarium* spp., and *Coccidioides* spp. Collectively, human fungal pathogens cause an estimated 6.55 million life-threatening infections each year, killing 3.75 million [(Denning 2024); gaffi.org]. The WHO report also highlighted the dangers of these infections, for example high case-fatality rates, limited antifungal classes, and an increasing case burden due to more frequent use of immunosuppression in the clinic.

In the laboratory, fungi serve as important model systems. The first description of the molecular underpinnings of the cell cycle relied in part on studies of baker’s yeast *Saccharomyces cerevisiae* (Hartwell et al. 1973, Nurse et al. 1976). The importance of telomeres and telomerase were first established using *S. cerevisiae* and the model protozoan *Tetrahymena* (Blackburn and Gall 1978, Szostak and Blackburn 1982). Molds like *Penicillium rubens* and *Aspergillus nidulans* have been biotechnological workhorses revealing huge potential for the isolation of novel secondary metabolites (Nielsen et al. 2017, Caesar et al. 2020), as well as providing the first molecular mechanisms for many noteworthy natural products.

In recent years, technologies to investigate nucleic acids have developed rapidly, particularly as we have transitioned from Sanger sequencing to Next-Generation Sequencing (NGS) and now even third/fourth generation sequencing approaches (Satam et al. 2023). Advances in mass spectrometry and electron microscopy have also fueled improved molecular characterization of nucleic acid structure, localization, and interaction partners. As tools to define nucleic acids have improved, so too has our understanding of their diverse cellular functions. Nucleic acids have complex lifecycles: DNA molecules are the genetic memory of the cell, but are also chemically modified, bound by proteins or nucleic acids, and packaged into massive molecular structures that undergo complex reorganization during replication and transcription (Minchin and Lodge 2019). Unlike DNA, RNA is typically single-stranded, allowing it to fold into complex secondary and tertiary structures that are also further diversified by chemical modifications. It is capable of catalytic activity on its own, for example in the ribosome, but is nearly always in association with proteins. The complexity of nucleic acids has limited their study in many cases to model fungi, but with the increased accessibility of advanced technologies in recent years, we are now beginning to ask questions in fungal pathogens that were previously not possible (Scazzocchio 2014).

In this review, we discuss the current state of technology as it relates to our understanding of the nucleic acid biology of fungal pathogens. We focus on recent advances in pathogenic fungi and bring in emerging technologies from related fields that might soon be applicable. We conclude with a discussion of the future of nucleic acid technologies, highlighting particularly exciting areas of development relevant to human health and society.

## Advances in genetic engineering are revolutionizing our molecular understanding of fungal pathogens

Fungi are invaluable model organisms in molecular biology, with functional analyses typically straightforward due to their compact genomes relative to Animalia. Targeted genetic manipulation of fungal genomes has traditionally relied on homologous recombination, a feature of DNA damage repair (Huang and Cook 2022). However, some fungi exhibit low rates of homology-directed repair (HDR), and instead, higher rates of repair via non-homologous end joining (NHEJ), which makes the specific exchange of a gene for a deletion cassette tedious and inefficient. Inactivation of NHEJ pathways through genetic deletion has been leveraged in some species to facilitate genetic manipulation through promotion of HDR (Ninomiya et al. 2004, da Silva Ferreira et al. 2006, Gandia et al. 2016, Matsumoto et al. 2021), but for many organisms, challenges remain (Arentshorst et al. 2012). The genetic manipulation of previously inaccessible fungal genomes, as well as targeted marker-free gene editing, was revolutionized with the introduction of CRISPR/Cas9, which overtook previously available but not very widely used technologies, such as zinc-finger nucleases (ZFNs) and transcription activator-like effector nucleases (TALENs) (Ding et al. 2019).

### CRISPR allows for efficient gene function analysis and diagnostics of fungal pathogens

**Figure 1:**
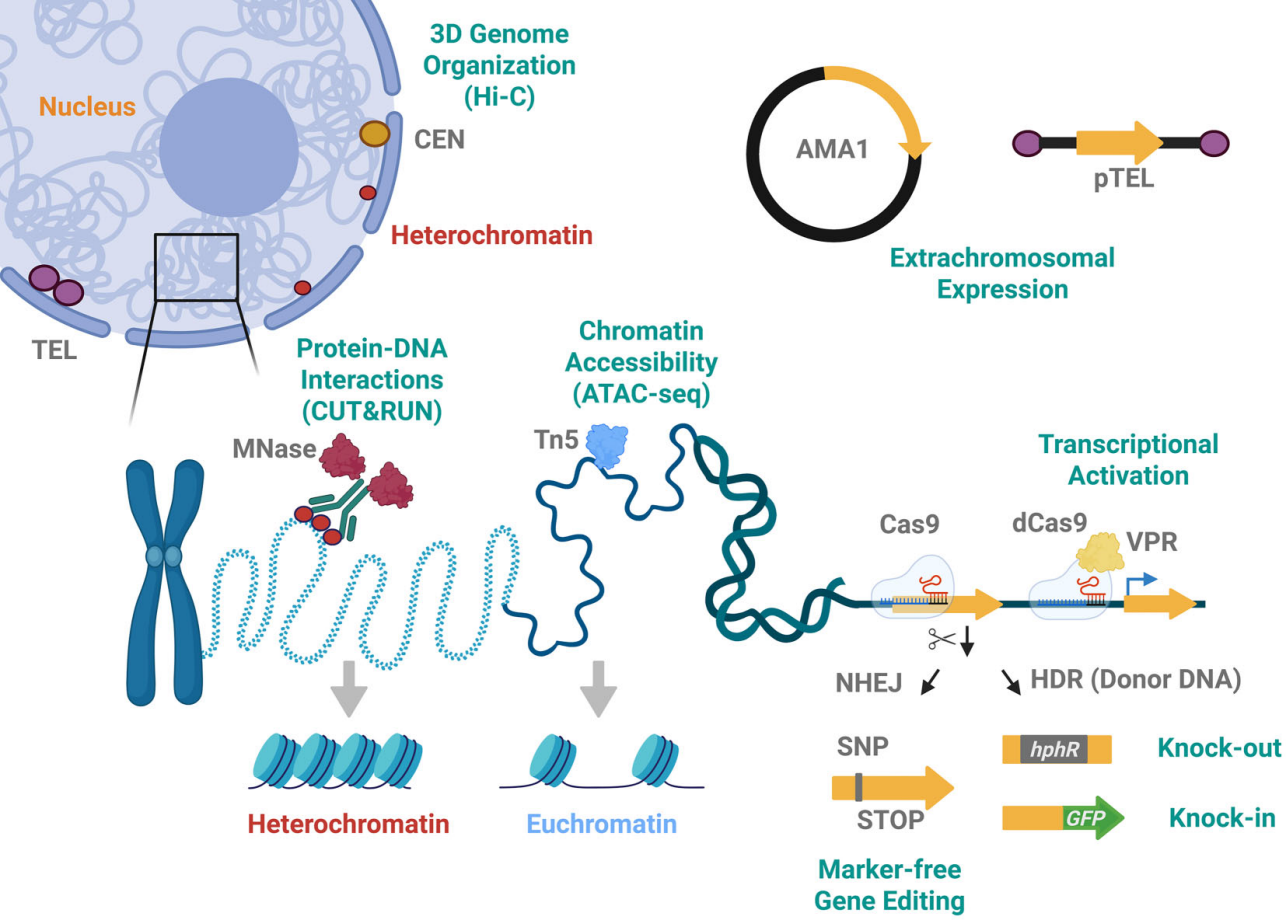
DNA technologies used to assess chromatin biology and manipulate fungal genomes. CRISPR/Cas9 can be employed both for marker-free gene editing, via the non-homologous end-joining (NHEJ) repair pathway, or marker-selected genetic modification, via homology-directed repair (HDR) and the supply of a donor DNA. Transcriptional activation of a gene of interest is possible under the use of a catalytically dead Cas9 (dCas9) variant fused to the tripartite activator VP64-p65-Rta (VPR). Additionally, extrachromosomal expression has been achieved with the AMA1 plasmid as well as linear telomere (TEL) vector. Concerning the chromatin methods described here, the assay for transposase-accessible chromatin with high-throughput sequencing (ATAC-seq) is employed for mapping chromatin accessibility, by tagmentation under the use of a hyperactive Tn5 transposase. Cleavage under targets & release using nuclease (CUT&RUN) is an improved chromatin immunoprecipitation method in which the protein-bound target DNA is released by micrococcal nuclease (MNase) digestion, mapping for example histone modifications. High-throughput chromosome conformation capture (Hi-C) quantifies genome-wide chromatin interactions, thereby allowing insights into fungal 3D genome organization. CEN, centromere; SNP, single-nucleotide polymorphism. Created in BioRender. Blango, M. (2026) https://BioRender.com/xlk78lk.

The discovery of the clustered regularly interspaced short palindromic repeats (CRISPR)/Cas9 (CRISPR-associated protein 9) system has transformed most fields, including the field of fungal genetics (Jinek et al. 2012). This technology exploits a bacterial defense system against phages. It relies on a single guide RNA (sgRNA) and the Cas9 protein, a type II RNA-guided endonuclease to recognize and cleave phage sequences, respectively (Jinek et al. 2012). The system has been reviewed extensively elsewhere (Adli 2018, Wang and Doudna 2023, Pacesa et al. 2024). In brief, the sgRNA is typically composed of a 20-nucleotide sequence known as the CRISPR RNA (crRNA), that is highly specific for the target sequence through the requirement for a neighboring protospacer adjacent motif (PAM), and a trans-activating crRNA (tracrRNA) that serves as the scaffold to bind Cas9 (Zhang et al. 2016, Asmamaw and Zawdie 2021). Directed by the sgRNA, the Cas9 protein acts as molecular scissors inducing a double-strand break at the DNA target site positioned three base pairs before the PAM sequence (Makarova et al. 2011). Gene editing through repair of the break can be done 1) marker-free via NHEJ, which is error-prone and frequently results in the random insertion or deletion of nucleotides, or 2) via HDR under the use of a template DNA with adjacent homologous sequences (Doudna and Charpentier 2014) (Fig. 1).

CRISPR/Cas9 has found multiple applications in fungal pathogens. In the opportunistic human pathogen *C. albicans*, codon-optimized *CaCAS9* and sgRNA cassettes delivered as DNA for transient expression were shown to yield successful gene deletion without the need for stable genome integration (Min et al. 2016). However, the mutation rate was not high enough for use in gene editing without selection (Ng and Dean 2017). Noteworthy, the diploid genome of *C. albicans* requires editing of both alleles for gene function analysis. An autonomously replicating plasmid expressing CaCas9 was generated so that an sgRNA can be introduced in one cloning step making this editing system functional in several *Candida* species (Lombardi et al. 2019). The implementation of a marker recycling strategy was shown to quicken the generation of multiple-mutant strains in *C. albicans* (Huang and Mitchell 2017). Additional advancement of the CRISPR system in *C. albicans* was achieved with CRISPR-GRIT, which expresses sgRNAs with integrated repair templates for a faster rate of gene deletions and multiplex gene knockouts (Cotter and Trinh 2024).

For *A. fumigatus*, an *in vitro* assembly method of dual Cas9-sgRNA ribonucleoproteins (RNPs) for targeted gene deletion was developed (Al Abdallah et al. 2017). This system overcomes the limitations of time-consuming DNA-based systems that require the construction of expression vectors and engineering of fungal strains to express Cas9 and sgRNA. Due to the transient expression of Cas9, the system aids in reducing off-targets, and is functional with micro-homology arms as little as 35 to 50 bp in the repair template DNA (Al Abdallah et al. 2017). This methodology enabled efficient gene deletion in the wild-type strain Af293 (74% efficiency using 50 bp homology arms), which normally exhibits low rates of HDR and calls for the use of the NHEJ-deficient Δ*akuB* background (Al Abdallah et al. 2017).

Wang et al. developed an optimized and rapid Cas9 RNP transformation protocol for genetic manipulation of *F. oxysporum*, an important trans-kingdom pathogen of plants and humans. They employed purified Cas9 tagged with the endogenous H2B nuclear localization signal for improved transient nuclear targeting of Cas9 (Wang et al. 2018). By applying a homology-independent targeted integration strategy, they inserted a large DNA fragment of about 8 kb at the sgRNA site, while flanked donor DNA was applied for the standard homology-dependent integration approach (Wang and Coleman 2019). Additionally, a versatile vector-based CRISPR/Cas9 system was used for efficient targeted knock-out, knock-in, and replacement strategies in multiple *Fusarium* species, including the accessory genome of *F. oxysporum*, which normally shows lower rates of HDR (Shinkado et al. 2022). This was possible using a U6 small nuclear RNA promoter for sgRNA expression (Shinkado et al. 2022).

CRISPR/Cas9 has been particularly transformative for difficult-to-manipulate fungal pathogens. For example, a vector-based CRISPR-Cas9 system was used in *Histoplasma* (Joehnk et al. 2023) and a plasmid-free system in *Mucor circinelloides* to create gene deletion strains (Nagy et al. 2017), overcoming long-standing issues of genetic manipulation in these organisms. Too numerous to list here, the CRISPR/Cas9 system has been leveraged to unravel attributes underlying gene regulation in a wide-variety of fungal pathogens, e.g. natural product biosynthesis as well as virulence mechanisms of fungal pathogens (Weber et al. 2017, Arai et al. 2021, Wang et al. 2021, Wassano et al. 2024).

The CRISPR/Cas technology is not only useful for gene editing but also for molecular diagnostics, as timely detection of an invasive fungal pathogen is essential in combating opportunistic infections. Investigations of CRISPR-based diagnostics in fungi have been extended from exciting advances with bacterial and viral pathogens (Gootenberg et al. 2017, Broughton et al. 2020). One such example comes from CRISPR-Cas12a systems, which use a single crRNA and the type V endonuclease Cas12a to cleave DNA substrates and recognize a short T-rich PAM (Rananaware et al. 2023). The authors leveraged *trans*-cleavage of a fluorophore and quencher (FQ) reporter by Cas12a to create the Split Activator for Highly Accessible RNA Analysis (SAHARA) system and demonstrate efficient detection of Hepatitis C virus RNA. A CRISPR-Cascade assay that integrates a positive feedback loop recently improved on the slow *trans*-cleavage of many Cas enzymes to create a rapid and sensitive detection system for viral and bacterial bloodstream infections (Lim et al. 2025). These intriguing technologies are also starting to be applied to detection of nucleic acids in fungal pathogens.

In 2024, an RPA-CRISPR/Cas12a technology for the rapid detection of *A. fumigatus* was developed (Lin et al. 2024). Recombinase polymerase (RPA) is a sensitive isothermal amplification technology that is widely used to function at low temperatures ranging from 37°C to 42°C, giving enough product within ∼10–30 min (Daher et al. 2016). The RPA-Cas12a-fluorescence method and RPA-Cas12a-LFS (lateral flow strip) can be used to display the results. Both platforms are highly sensitive, specific, and consistent, making it a possible diagnostic platform in clinics (Li et al. 2022). In addition, this highly sensitive system can detect up to 10 fg of target DNA of banana-infecting *F. oxysporum* f. sp. *cubense* TR4. Remarkably, it is even possible to observe infected banana samples with the naked eye using LED blue light transillumination (Matthews et al. 2025). In *C. albicans*, a chitin affinity-magnetic separation and RPA-CRISPR/Cas12a one-pot detection system was capable of detecting 30 CFU/mL of *C. albicans* spiked into blood or bronchoalveolar lavage in 2.5 h making it highly sensitive as compared to microscopy-based staining methods (Shen et al. 2024). It is important to note that although the improved detection sensitivity is promising, CRISPR-based diagnostics remain laborious and subjective in their interpretation due to the ubiquitous nature of many fungal pathogens. Taken together, the advancement of the CRISPR technology has indeed accelerated our understanding of gene function and provided an opportunity for novel diagnostics of fungal pathogens.

### AMA1, telomere vectors, and CRISPRa are useful tools for the targeted activation of fungal gene expression

Understanding the activation of gene expression in fungal pathogens is vital to grasping the molecular mechanisms underlying their pathogenicity, adaptation to their environment, and fungal-host interaction. AMA1 (autonomously maintained in *Aspergillus*) (Fig. 1) is an autonomously replicating plasmid isolated from a genomic library of *A. nidulans*, which harbors an inverted repeat fostering extrachromosomal replication (Aleksenko and Clutterbuck 1996). AMA1-based expression offers advantages for (heterologous) expression in *Aspergillus* and *Penicillium* species, such as moderate transformation efficiency and robust expression from the multicopy vector (Aleksenko and Clutterbuck 1997); however, in practice the plasmid can be quickly lost, limiting appeal. Compared to AMA1, single-copy integration with technologies like CRISPR or Cre-lox recombinase can result in lower expression profiles, despite improving construct stability (Roux and Chooi 2022). Notably, expression levels of a single copy gene can be dramatically increased using optimized variations of strong constitutive promoters, such as *tef1INp* (Yuan et al. 2025). Recently, the stability of the AMA1 vector was improved, yielding next-generation AMA1-based plasmids, by fusing degradation tags to the auxotrophic marker (Roux et al. 2024). An interesting report revealed the successful cloning of all 56 secondary metabolite clusters from the opportunistic human pathogen *Aspergillus terreus* into a self-replicating fungal artificial chromosome (FAC) harboring the *Escherichia coli* F plasmid origin and the *Aspergillus* AMA1 replication element for heterologous expression in *A. nidulans*. This approach allowed for the identification of the *A. terreus* astechrome cluster (Bok et al. 2015). Similarly, the expression of a FAC encoding the *Pseudogymnoascus destructans* squalene synthase Sqs in *A. nidulans* gave rise to the isolation of three new nidulene natural products (Park et al. 2024).

To address low transformation yields using AMA1 in the gray mold *B. cinerea*, an impressive study was conducted by applying RNP-based CRISPR/Cas9 and co-transforming a selectable and transient telomere vector (Leisen et al. 2020). The telomere vector (Fig. 1) contains human telomere sequences, and while it is amplifiable as a plasmid in *E. coli*, it linearizes into a non-integrative mini-chromosome in *B. cinerea* and likely other filamentous fungi. Consequently, the loss of selection pressure results in telomere vector curing. With this method, a high number of marker-free edited transformants were achieved in each experiment (Leisen et al. 2020). In the rice blast fungus *Magnaporthe oryzae* (syn. *Pyricularia oryzae*), the essential gene *MoPKC* (protein kinase C) was deleted using a telomere vector carrying a copy of this gene. Remarkably, transformants without successful *MoPKC* deletion lost the telomere vector under non-selective conditions, whereas the ones with successful deletions had the vector retained, demonstrating the essentiality of the gene (Wirtz et al. 2024). The importance of *MoPKC* in many cellular processes, including virulence and antifungal drug tolerance, and the fact that its deletion strongly affects vegetative growth (Penn et al. 2015), somewhat complicates interpretation. Additional work will be required to fully understand the limitations of such approaches in relation to gene function.

The CRISPRa system uses a nuclease-dead dCas9 protein fused to a transcriptional activator and is advantageous in the generation of genetic overexpression systems, especially in the production of economically vital metabolites and new bioactive molecules (Donohoue et al. 2018, Roux et al. 2020, Jiang et al. 2021). An sgRNA is used to direct the activator complex to the promoter sequence of the gene of interest (Gervais et al. 2023). Based on the CRISPRa method, dCas9 fused to a tripartite activator VP64-p65-Rta (VPR) (Fig. 1) and encoded on a non-integrative AMA1-vector allowed for the successful activation of the transcriptionally silent *P. rubens* macrophorin biosynthetic gene cluster and production of antimicrobial macrophorins (Mozsik et al. 2021). Overall, technologies like AMA1, telomere vectors, and CRISPRa allow for activation of gene expression to study gene function in fungal pathogens or to (heterologously) express biosynthetic gene clusters.

## Emerging technologies are unraveling chromatin dynamics in fungal pathogens

Several emerging technologies for the elucidation of chromatin landscape and chromosome organization are allowing powerful insights into how fungal pathogens regulate gene expression and potentially adapt to diverse host environments. Chromatin is a complex of DNA and proteins (primarily histone octamers), both of which can be chemically modified to allow for higher-order gene regulation and to influence local transcription factor-mediated gene regulation (Collemare and Seidl 2019). While loosely packed euchromatin is stably expressed under diverse environmental conditions and ensures the maintenance of basic cellular function, regions of densely packed heterochromatin are associated with gene repression (Freitag 2017). For the latter, one can then differentiate between constitutive heterochromatin at centromeres and telomeres destined for long-term gene silencing and facultative heterochromatin that is more readily activated upon certain environmental cues. In fungal pathogens, virulence genes and secondary metabolite gene clusters are typically found within regions of facultative heterochromatin (Wiles and Selker 2017, Janevska et al. 2026). This part sheds light on tools for mapping histone modifications, chromatin accessibility, DNA-binding proteins, and impressive approaches that can reveal the three-dimensional (3D) genome structure of fungi.

### ATAC-seq is a powerful tool for genome-wide mapping of chromatin accessibility

Several chromatin accessibility profiling methods have been developed, such as deoxyribonuclease I hypersensitivity sequencing (DNase-seq) (Boyle 2008 ), formaldehyde-assisted isolation of regulatory elements sequencing (FAIRE-seq) (Giresi 2007, Giresi 2009), micrococcal nuclease digestion with deep sequencing (MNase-seq) (Albert 2007 ), methylated DNA immunoprecipitation sequencing (MeDIP-seq) (Kelly 2012 ), and the assay for transposase-accessible chromatin with high-throughput sequencing (ATAC-seq). ATAC-seq is widely used for identifying open, nucleosome-free, and nucleosome-bound chromatin as well as (motifs of) DNA-binding proteins (Buenrostro 2013 ). In comparison with other methods, such as the original DNase-seq and MNase-seq, ATAC-seq is a fast, efficient, and robust method for chromatin profiling as library preparation is fast and low input material is required (Grandi 2022 ).

ATAC-seq (Fig. 1) utilizes hyperactive Tn5 transposase (Goryshin 1998 ) to simultaneously fragment and ligate adapters in regions of open chromatin for library preparation and high-throughput sequencing using a procedure known as ‘‘tagmentation” (Grandi 2022). Reads from high-throughput sequencing are mapped to a reference genome, so that high read coverage indicates accessible chromatin. In *Aspergillus niger*, ATAC-seq led to the identification of basic leucine zipper (bZIP) transcription factors that are linked with different stress responses such as amino acid starvation, oxidative stress, and sulfur metabolism (Huang 2021 ). The study additionally highlighted that comparative genome analysis is possible with ATAC-seq, in this case comparing *A. niger* with *Aspergillus oryzae* (Huang 2021).

For *C. albicans*, ATAC-seq has been effectively used to gain insights into chromatin accessibility and gene expression in response to oxidative stress (Jenull 2020 )*. De novo* motif analysis revealed that upon H_2_O_2_ treatment, open chromatin regions showed enrichment for binding sites of Cap1p, a transcription factor known to be associated with oxidative stress pathways (Wang 2006). Moreover, two transcription factors of the wheat pathogen *Fusarium graminearum* FgAreB and FgSnf5, were found to be important in collaboratively maintaining chromatin accessibility in response to nitrosative stress (Jian 2021 ). ATAC-seq analysis in *Penicillium expansum* revealed that deletion of the bZIP transcription factor gene *PeAtf1* increased tolerance to oxidative, cell wall, and membrane stresses, and could be associated with virulence in apple fruits (Wang 2025). Collectively, ATAC-seq is a useful tool for identifying (infection-related) transcription factors as well as open vs. closed chromatin states in response to various treatments.

### CUT&RUN and CUT&tag technologies elucidate protein-DNA interaction dynamics

Conventional chromatin immunoprecipitation (ChIP) has been a common and practical solution for studying the interactions of proteins or protein complexes with chromosomal DNA *in vivo* (Landt et al. 2012, Wiehle and Breiling 2016). The enrichment of a (tagged) transcription factor, cofactor, or a specific histone modification is achieved by crosslinking of DNA-protein-complexes, chromatin shearing, and subsequent use of a specific antibody, thereby making it possible to target essentially any chromosomal protein (Boedi et al. 2012). Later, the implementation of ChIP-sequencing (ChIP-seq) aided in the genome-wide mapping of DNA-binding proteins or histone modifications (Park 2009). Several studies have used ChIP-seq on fungal pathogens such as *A. fumigatus, C. albicans, M. oryzae, Fusarium fujikuroi*, and more (Chung et al. 2014, Janevska et al. 2018, Price et al. 2019, Zhang et al. 2021). Some limitations of this traditional method include lack of resolution (Wiehle and Breiling 2016), length and cost of the experiment, high background noise, and the requirement of large sample inputs (Zhu et al. 2019, Qasim et al. 2022). Combination of ChIP-seq with RNA-seq approaches has further enriched our understanding of gene regulation, as with the discovery of an interconnected network regulating biofilm formation in *C. albicans* (Nobile et al. 2012).

Cleavage under targets & release using nuclease (CUT&RUN) (Fig. 1) was developed in 2017 to overcome some of the previous limitations (Skene and Henikoff 2017). As compared to classical ChIP, there is no requirement for crosslinking or chromatin shearing. Isolated intact nuclei are incubated with a specific antibody linked to protein A/G-MNase (Meers et al. 2019). The protein-bound target DNA is released by MNase digestion, allowing for purification and sequencing (Skene and Henikoff 2017). An advantage of this method is a higher signal-to-noise ratio, which aids in the detection of new genomic elements (Skene et al. 2018). Moreover, lower amounts of starting material are necessary compared to classical ChIP, making this method more cost-effective (Qasim et al. 2022).

CUT&RUN has not been widely applied to study DNA-protein interactions in fungal pathogens so far. For *C. albicans*, a CUT&RUN protocol was established for the genome-wide analysis of transcription factor-DNA binding interactions, using NDT80-GFP as a proof-of-concept (Qasim et al. 2022). A detailed description of the CUT&RUN protocol with all important steps was outlined, such as epitope-tagging of transcription factor-coding genes, library preparation, and sequencing, along with a computational pipeline for CUT&RUN data analysis (Qasim et al. 2022).

Although CUT&RUN generates high-quality results and needs a small amount of starting sample, it is not ideal for single-cell profiling applications due to the release of the fragments into the supernatant (Zheng et al. 2017). To overcome this limitation, cleavage under targets and tagmentation (CUT&Tag) was developed, applying a so-called transposome, i.e. a hyperactive Tn5 transposase-protein A fusion protein pre-loaded with DNA-sequencing adapters (Reznikoff 2003). In CUT&Tag, the protein of interest is bound by a primary antibody, after which a secondary antibody enhances binding of the Tn5 fusion protein. Consequently, activated by Mg^2+^, Tn5 integrates adapters at DNA-protein binding sites, which are enriched by PCR during library preparation (Fu et al. 2024).

In *F. graminearum*, CUT&Tag-seq was performed for efficient mapping of histone modifications under secondary metabolite-inducing conditions, along with RNA-seq, ATAC-seq, and Hi-C-seq (see next section) (Shao et al. 2024). The authors reported a chromatin structure designated “jet-like domains,” which correlates with active transcription and histone acetylation, primarily driven by the Gcn5 histone acetyltransferase complex (Shao et al. 2024). This powerful study highlights the relationship between epigenetic modifications, chromatin architecture, and gene regulation.

### Hi-C sheds light on fungal 3D genome organization

The 3D genome structure of the fungal genome plays a vital role in chromosomal mechanisms such as transcriptional regulation, DNA repair, and DNA replication (Torres et al. 2023). High-throughput chromosome conformation capture (Hi-C) quantifies genome-wide chromatin interactions, allowing for the determination of interactions between nearby and distant regions within the same chromosome or among chromosomes. After chemical crosslinking to freeze a snapshot of chromosome architecture, cells are lysed and the DNA is subjected to digestion using specific restriction enzymes. The ends of the resulting fragments are marked with biotin, after which fragments with 3D proximity are ligated using a method called dilution ligation that promotes intramolecular ligation. After reverse crosslinking, ligated junctions are enriched from the soluble DNA and purified using streptavidin beads. Finally, high-throughput sequencing of these chimeric molecules allows for the genome-wide mapping of pair-wise chromatin interactions and the determination of relative proximity (Seidl et al. 2020, Lafontaine et al. 2021).

In fungi, nuclear genome organization can be explained with the following subnuclear structures of hierarchically increasing size: chromatin loops (such as enhancer-promoter contacts), topologically associated domain (TAD)-like structures, and the Rabl chromosome conformation (named after Carl Rabl). The latter is characterized by centromeres and telomeres associating with opposite sides of the nuclear envelope, which allows for the aggregation of euchromatin vs. heterochromatin compartments within the nucleus (Fig. 1), critical for proper genome function (Torres et al. 2023). For metazoans, TADs are well-characterized chromosomal compartments, which are typically maintained by the CCCTC-binding factor (CTCF), condensin, cohesin, and histone modifications for stability (Rajderkar 2023 ). By definition, TADs are linear units of chromatin that are folded into distinct 3D structures (Ong and Corces 2014, Ciabrelli and Cavalli 2015). As CTCFs have not been described in fungi, these compartments are often referred to as TAD-like (Torres et al. 2023).

Elucidation of the 3D genome organization of the broad-range vascular wilt pathogen *Verticillium dahliae* further characterized so-called adaptive genomic regions (AGRs), which harbor virulence-associated genes, and interestingly, show high chromatin accessibility while being transcriptionally repressed under laboratory conditions by facultative heterochromatin (Cook et al. 2020). Hi-C revealed that AGRs physically co-localize within the nucleus (Torres et al. 2024). TADs were identified as short-range DNA interactions with gene-rich boundaries, the latter being characterized by reduced levels of gene expression compared to genes within TADs. Notably, TADs within the core genome were more clearly “insulated” compared TADs within AGRs (Torres et al. 2024). Furthermore, using Hi-C, a chromosome-level assembly of the rust fungus *Puccinia striiformis* f. sp. *tritici* was generated, and the genome was analyzed concerning its 3D organization (Xia et al. 2022). Strikingly, no chromosome compartmentalization became obvious in this fungus. Moreover, the 3D genome organization of two developmental stages revealed that gene regulation does not depend on the alteration of genome organization (Xia et al. 2022).

In a study on neocentromeres in *C. albicans*, Hi-C revealed that neocentromeres clustered together with native centromeres (Burrack et al. 2016). This implies that the remodelling of 3D nuclear architecture is mediated by a new centromere playing an evolutionary role in adaptation or speciation (Amor and Choo 2002).

As indicated above, besides studying 3D chromosome architecture, Hi-C can also be used for improving genome assemblies of fungal pathogens. For example, a gap-free genome assembly of corn-infecting *Fusarium verticillioides* was generated using PacBio HiFi and Hi-C sequencing data, highlighting several chromatin loop and TAD-like structures (Yao et al. 2023). Additionally, the combination of PacBio HiFi and Hi-C sequencing technologies led to a gap-free genome assembly of the *Arabidopsis*-infecting *F. oxysporum* strain *Fo* 5176 (Wang et al. 2024).

In conclusion, emerging technologies such as ATAC-seq, CUT&RUN, CUT&Tag, and Hi-C are excellent tools for studying the chromatin landscape of fungal pathogens. We are awaiting the application of these methods in more fungal models to be able to evaluate their general applicability and contribution to our understanding of fungal chromatin biology. Additionally, the change of the chromatin landscape during the host-pathogen-interaction remains elusive at large and of great interest to the community.

## Technologies to assess RNA sequence, structure, and intermolecular interactions have improved dramatically over the past decade

Our capacity to study the RNA macromolecule has improved in parallel to our advances in investigating DNA in fungi, but our understanding of RNA regulation in fungal pathogens lags behind that of model fungi. The biggest advances in studying RNA have again largely centered on improvements in high-throughput sequencing technologies, biochemical techniques, and bioinformatic workflows. In this section, we will discuss the aspects of RNA technologies that have advanced regarding determination of RNA processing, localization, and intermolecular interactions and incorporate some discussion of emerging techniques so far not widely reported in fungal pathogens.

### RNA technologies improve our ability to comprehensively define the lifecycle of each transcribed RNA in a cell

**Figure 2:**
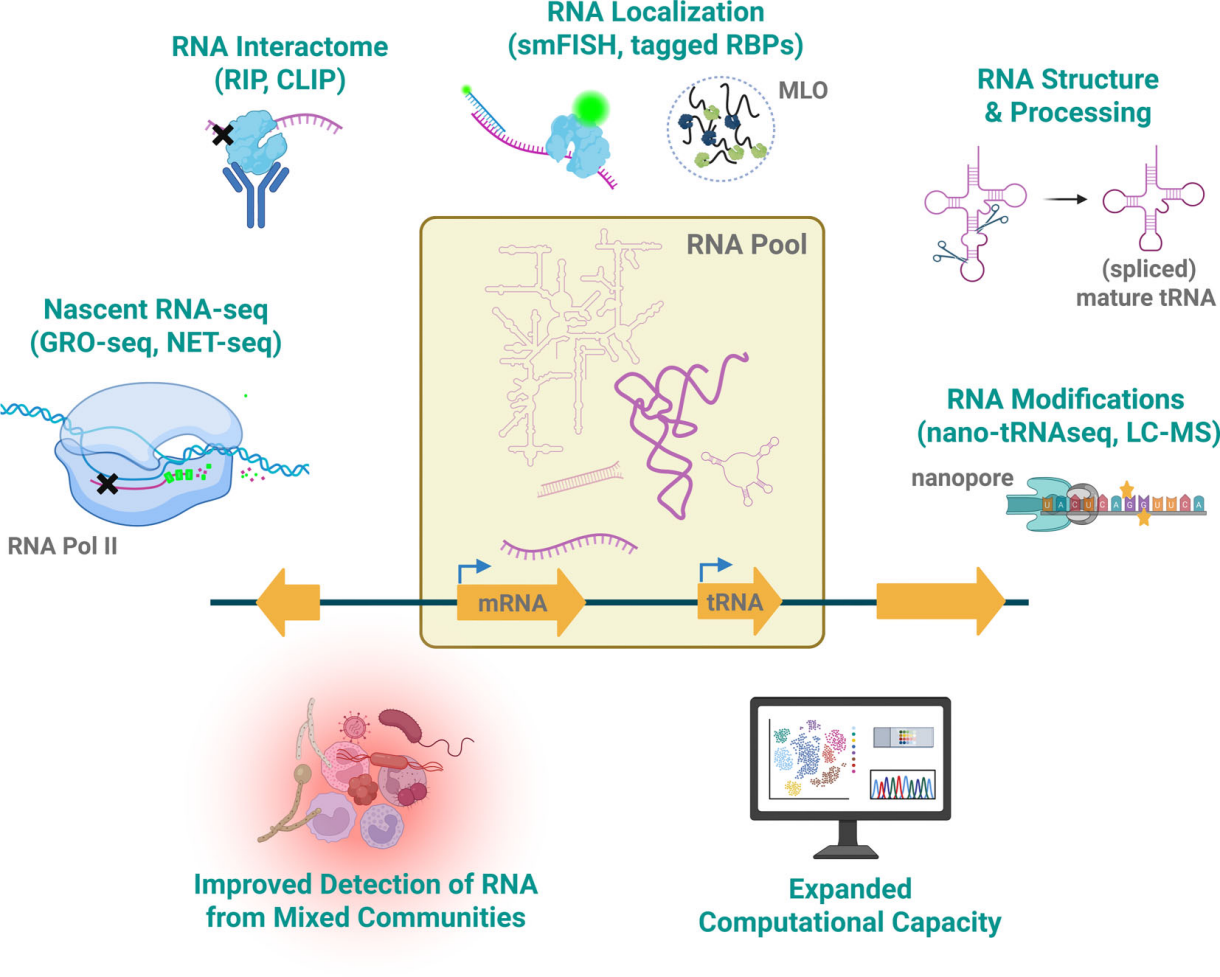
RNA technologies offer unprecedented possibilities for discerning RNA regulation in fungal pathogens. The cellular RNA pool is a heterogenous population of RNAs with diverse functions. Technologies now exist to assess the RNA macromolecule at each step of the lifecycle, from initial transcription of the nascent RNA, using techniques like global run-on sequencing (GRO-seq) or native elongating transcript sequencing (NET-seq), to the localization of the RNA to its final functional destination. Techniques like RNA immunoprecipitation sequencing (RIP-seq) and cross-linking immunoprecipitation-high-throughput sequencing (CLIP-seq) are used to assess RNA-protein interactions, whereas a series of biochemical approaches have emerged to assess RNA structure and modification. Improved sensitivity of RNA-seq approaches and computational capacity has allowed for refined detection of RNA in microbial communities and more robust statistical analysis of differential RNA abundance, respectively. MLO, membrane-less organelle. Created in BioRender. Blango, M. (2026) https://BioRender.com/y9bvh0k.

RNA molecules are transcribed by RNA polymerases, and the resulting immature RNA is known as the nascent RNA prior to processing. This unprocessed RNA is of great interest to researchers, as it informs transcription dynamics, RNA processing, and relevant regulatory elements/factors. There are three main types of nascent RNA-seq, although numerous variations exist: 1) GRO-seq (global run-on-seq) (Fig. 2) relies on incorporation of labeled nucleotides (typically 5-bromouridine-5′-triphosphate or 4-thiouridine) into actively transcribed genes. Subsequently labeled RNA can be specifically selected by affinity purification. PRO-seq (precision nuclear run-on sequencing) offers an improvement, where biotin-NTP is used during the nascent RNA enrichment process. 2) In caRNA-seq (chromatin-associated RNA-seq), chromatin-bound RNA is enriched via high-salt washes. 3) NET-seq (native elongating transcript-seq) is a derivative of RIP-seq (RNA immunoprecipitation-seq) (Fig. 2), where RNA is crosslinked to proteins and then the RNA-polymerase of interest is immunoprecipitated. All of these have their individual pros and cons and choosing the right method for answering a specific question is essential (reviewed in Wissink et al. 2019).

Within the fungal kingdom these methods have been mainly used in the model organisms *S. cerevisiae* and *Schizosaccharomyces pombe*. GRO-seq was used in *S. cerevisiae* to show that Clp1 (part of the Cleavage Factor IA complex) is important for efficient termination of highly transcribed genes and also termination of promoter antisense RNA, thereby determining promoter directionality (Al-Husini et al. 2017). These approaches work well in controlled laboratory environments with ample material but are challenging in fungal samples from patients where material is limited. A confounding factor in both cases is the fungal cell wall, which may hinder entry of modified nucleotides into the cytoplasm.

With the development of csRNA-seq (capped small RNA-seq) in 2019 (Duttke et al. 2019), some of these barriers were overcome. csRNA-seq enriches for 5′-capped small RNAs from total RNA by gel electrophoresis; a subsequent phosphorylation and degradation of uncapped 5′-ends decreased false positives. This method can be used to determine transcriptional start sites for a range of RNAs, but it also detects rapid transcriptional changes with an order of magnitude more sensitivity than standard RNA-seq. This technique was used to identify transcription start regions (TSR) in *Coccidioides immitis* to reveal differential usage of TSRs between growth stages (Duttke et al. 2022). Studies of transcriptional regulation may also lead to promising new drug targets. For example, an enrichment in TSRs associated with the binding motif for the WOPR (white-opaque switching regulator) transcription factor family was observed in the virulent spherule stage. As the WOPR-motif is fungal-specific (Zhang et al. 2014) and binding of the WOPR transcription factor (CIMG_02 671 in *C. immitis*) is essential for the onset of valley fever, WOPR transcription factor inhibition may be a promising therapeutic approach.

Other specialized forms of RNA-seq have defined basic parameters of RNA molecules in *C. neoformans* and *A. fumigatus* as well, as with 5′- and 3′-end sequencing approaches (Wallace et al. 2020, Schrettenbrunner et al. 2026). These approaches levy the poly(A) tail for selection of intact mRNAs, and then rely on enzymatic processing to enrich for 5′ or 3′ ends. In another recent study on *Cryptococcus*, a gene knockout library was used to identify a transcription factor, Tur1, that appears important for dictating alternative 5′-end selection (Dang et al. 2024). Integration of these end-mapping approaches with gene knockout libraries or other -omics approaches has been particularly powerful in revealing novel biology. In the future, more in-depth analyses in infection contexts will hopefully determine whether the transcriptional landscape changes in harmony with the host response.

### The interplay of RNA structure and modifications in fungal pathogens

The single-stranded nature of transcribed RNA facilitates complex folding into secondary and even tertiary structures. The easiest example of RNA structure to envision is the tRNA molecule, which forms an iconic cloverleaf secondary structure with multiple hairpin loops and an L-shaped tertiary structure created from numerous intermolecular contacts. Adding to the complexity, tRNA nucleotides are heavily chemically modified, with consequences for translational speed and fidelity. The vast majority of work studying the chemical modifications [more than 170 known modifications (Zhang et al. 2022)] and structures of RNA in microbes has been performed in model bacteria and fungi, but a few studies have started to carry technical advances into pathogens. The central role of tRNA modifications in tuning translation can be assessed using techniques like the well-established ribosome profiling. Although not exactly new, ribosome profiling has rarely been applied to fungal pathogens, in part due to complicated sample preparation. In *C. albicans*, ribosomal profiling was used to understand the effect of t^6^A RNA modification-loss on translation, and even to reveal the contribution of changes in ribosome occupancy during biofilm formation or other morphotype changes (Mundodi et al. 2021, Bottcher et al. 2024, Mundodi et al. 2025). We expect to see this technique adopted more widely in the future.

Oxford Nanopore direct RNA-sequencing is another emerging technique to assess full-length RNAs and associated modifications. Although the analysis of RNA modifications remains challenging, this technique was recently applied to study the *C. albicans* mitochondrial transcriptome *in flagranti*, where it provided the first identification of mitochondrial tRNA modifications in the organism (Piatkowski et al. 2024). Other applications of direct RNA-seq in pathogenic fungi include a global study of RNA methylations in *C. albicans* (Fan et al. 2021) and a new genome annotation of *Candida nivariensis* (Watson et al. 2024). A newer variation called nano-tRNAseq was recently used to investigate the modifications on *A. fumigatus* tRNAs (Bruch et al. 2025) (Fig. 2). Nano-tRNAseq facilitates the quantification and detection of modifications on highly structured tRNA molecules by reverse transcription of a cDNA and adapter that serves as a splint to aid in tRNA linearization and loading into the nanopore (Lucas et al. 2024). Advances in nano-tRNAseq are appearing rapidly, with improved computational approaches taking the lead. These allow for better quantification of tRNAs, assessment of tRNA acylation status (White et al. 2025), and even improved detection of *de novo* RNA modifications (White et al. 2024, Rubsam et al. 2025). The hope for the future is that even in the absence of a reference with known modifications, scientists will be able to assess the entire repertoire of modifications on an RNA molecule without prior knowledge of the organism, facilitating the full description of RNA modifications often designated as the epitranscriptome [discussed in (National Academies of Sciences 2024)]. The applications of this technology are only just being realized, but already there is a lot of “buzz” about the potential of using approaches like Oxford Nanopore Sequencing for diagnostic purposes.

We believe there is therapeutic potential in basic research into fungal pathogens. For example, fungal tRNA biology is quite unique in that 1) their tRNA genes contain a higher proportion of introns than plants or metazoans (Stuart et al. 2025) and 2) the splicing mechanism of fungal tRNAs is a three-step process rather than the two-step process of metazoans (Gerber et al. 2022) (Fig. 2), making tRNA splicing a proposed target for future antifungal development. Point 1 opens a new layer of regulation with the excised tRNA-introns functioning as gene regulatory sRNAs in *S. cerevisiae* (Nostramo et al. 2025), although further work is needed in fungal pathogens. The essential enzyme Trl1 that ligates the tRNA exons together is not found in metazoans (Phizicky et al. 1992), which has led researchers to suggest it as a potential drug target (Banerjee et al. 2019). tRNAs can also serve as the source of tRNA-derived RNAs (tDR), which are part of the sRNA repertoire. In *F. graminearum* these have been shown to be dependent on the canonical RNAi machinery (dicer and argonaute proteins) for their formation (Werner et al. 2021), and tDRs have been shown to be readily secreted by the basidiomycete *Ustilago maydis* (Yoshimoto et al. 2022). As multiple studies have shown that phytopathogenic fungi and plants bombard each other with secreted sRNAs (Wang et al. 2016, Cheng et al. 2023), the abundant tDRs might also contribute to communication across kingdoms in exciting ways. Sequencing of tDRs has proven to be technically challenging due to frequent modification and unconventional RNA-ends of a 2′,3′-cyclic phosphate at the 3′-end and a 5′-OH, which make cDNA-synthesis and conventional adaptor ligations inefficient. tDR-seq approaches that use enzymatic removal of key modifications and end repair strategies have now been applied in select organisms (Pan et al. 2026), but the full importance of these fragments remains under investigation. It is worth mentioning that fungi also encode microRNA-like RNAs (milRNAs), but these are generally poorly conserved and understood, as a computational global reanalysis of deep sequenced sRNA from 41 fungal species recently showed (Johnson et al. 2022). In fact, we know almost nothing of RNA structure in fungal pathogens, despite a few studies suggesting interesting diversity in this area (Rivas 2021, Chorostecki et al. 2024). How unique fungal RNA structures might be targeted therapeutically is only just starting to be considered.

### RNA is never alone in the cell: techniques to study RNA-binding proteins and localization

The single-stranded nature of RNA allows it to form a diversity of structures, but also facilitates abundant interactions with other macromolecules, particularly proteins. Proteins contribute to RNA biology in nearly every way, influencing structure, stability, function, and more. It is then not surprising that techniques to advance the study of proteins have also improved our ability to define RNA. For example, increased sensitivity of LC-MS/MS-based proteomics approaches have improved the resolution of RNA-binding proteins (RBPs) in general and allowed for more delicate dissection of downstream functionality. Techniques like cryo-electron microscopy have built on decades of x-ray crystallography and nuclear magnetic resonance studies to improve interactions of RNA and proteins at the structural level.

Recently, several varieties of immunoprecipitation methods in combination with RNA have been developed to study RNA-protein interactions. The most notable method is likely cross-linking immunoprecipitation-high-throughput sequencing (CLIP-seq) (Fig. 2). It relies on the same principles as ChIP-seq, namely crosslinking (UV instead of formaldehyde), immunoprecipitation of the protein of interest, and nucleic acid isolation. Several modified variations of this protocol exist, each with a tweak to account for specific needs [reviewed in (Xiang et al. 2024)]. To date, CLIP-seq has not been performed in pathogenic fungi, likely due to challenges associated with UV-crosslinking in fungi and a limited availability of antibodies against fungal RBPs, necessitating additional genetic manipulation to create and validate epitope-tagged strains. In *F. graminearum*, RIP-seq (RNA immunoprecipitation) (Fig. 2), which does not rely on crosslinking and returns only RNA fragments protected by proteins through an RNase digest, was used to study a novel splicing factor (FgRbp1). Together with a component of the spliceosome (U2AF23), FgRbp1 influenced the splicing efficiency of 47% of intron-containing genes, including genes important for sexual reproduction and wheat infection. As CLIP- and RIP-seq have been extensively used in *S. cerevisiae*, it seems only a matter of time before these techniques are optimized to shed light on pathogenic strategies.

RNA localization is a key driver of function, and a feature that can be mediated by both intrinsic RNA structure and protein binding partners. Unfortunately, determination of RNA localization in fungi is often challenging, again due to poor permeability of reagents through the fungal cell wall. Older studies in *C. albicans* provided strong hints of the potential impact of RNA localization, for example by following the *ASH1* mRNA trafficked by the She3 system using fluorescent in situ hybridization (FISH) to reveal mislocalization upon *SHE3* deletion (Elson et al. 2009). Further refinement of such tracking systems resulted in single-molecule FISH (smFISH) (Fig. 2) in *S. cerevisiae* (Tutucci and Singer 2020), which has now even been applied to *C. albicans* to localize RNAs in yeast, pseudohyphae, and hyphal cells (van Otterdijk et al. 2024). Although direct visualization and tracking of RNA is preferable, indirect approaches using tagged RBPs or reporter assays are more common (Fig. 2). In *U. maydis*, such a study was performed to track the RNA binder Rrm4, which supports localization of mRNA to the extending hyphal tip (Devan et al. 2024). Such an approach, combined with targeted mutagenesis, revealed a novel interaction motif in Rrm4 involved in endosomal mRNA transport. An exciting example of an RNA trafficking reporter comes from the study of interkingdom communication, where a reporter system established in the plant host *Arabidopsis thaliana* was used to detect trafficking of small RNAs from *B. cinerea* via activation of a fluorescent marker in the recipient cell (Cheng et al. 2023).

An emerging aspect of biological research is the membrane-less organelle (MLO) (Fig. 2). These are locations within the cell that form a gel-like structure and accumulate certain macromolecules, which often serve as “mini-bioreactors”[reviewed in (Staples 2023)] . The best studied examples for these are stress granules, where heat shock- or chaperone-encoding mRNAs are stored and released upon stress, and processing bodies (P-bodies), which form upon ribosome stalling to facilitate storage or mRNA decay. These examples hint at the important role of RNA in MLO biology. The plant pathogen *Ashbya gossypii* uses condensates to accumulate *Bni3* and *Spa2* mRNAs with the help of Whi3 at the site of new hyphal branchpoints for efficient actin assembly (Lee et al. 2015). In a similar mechanism, Whi3 also forms condensates with *Cln3* mRNA close to the nucleus to regulate asynchronous nuclear division (Lee et al. 2013). Both studies in *A. gossypii* used smFISH and RIP to prove localization and RBP interaction. In the human pathogen *C. neoformans*, MLOs were found to contribute to transcriptional regulation of Znf2, a regulator of the yeast-to-hyphae transition necessary for mating, via a feedback loop with the Ref1 protein (Glueck et al. 2024). Under standard conditions, Ref1 binds to the promoter of Znf2, inhibiting its transcription. Upon a mating signal, Znf2 binds to the promoter of Ref1 and enforces the utilization of an alternative transcription start site, resulting in a transcript encoding a protein isoform that is prone to form MLOs. This leads to sequestration of Ref1 away from the Znf2 promoter and facilitates higher transcription. It seems likely that such regulatory features are ubiquitous and as the tools to study MLOs develop, we are likely to see improved understanding of this intriguing biophysical feature of cells.

### Improved sensitivity has facilitated simultaneous detection of host and pathogen RNA

RNA-seq is clearly a pervasive and powerful tool to study transcriptional regulation, with recent iterations advancing our ability to detect not only abundant transcripts but rare molecules as well. This is exemplified by assessment of host-microbe interactions, where increased sensitivity and sample preparations has allowed for successful application of both dual and even triple RNA-seq, e.g. from a host and two microbes (Fig. 2).

Dual RNA-seq is now regularly attempted in a variety of systems. For example, assessment of the interaction of the insect-pathogenic fungus *B. bassiana* with greater wax moth larvae, *G. mellonella*, during infection revealed new gene modules as signatures of different infection stages using 76 deep-sequenced samples (Huang et al. 2023). A separate time course RNA-seq experiment of *B. bassiana* uncovered a set of transcription factors that are upregulated once the hyphae has passed the insect cuticle (Deng et al. 2024). At the center of this network, the transcription factor BbHCR1 was found to control virulence by influencing the expression of genes responsible for the morphological transition and the production of secondary metabolites.

The dual RNA-seq approach has also been applied to plant fungal pathogens, for example *Fusarium virguliforme*, which can interact with a wide range of hosts. Interestingly, it is highly virulent in some plants (e.g. soybeans), while infection is almost symptomless in others (e.g. maize) (Kolander et al. 2012). An RNA-seq time course of infected roots using both types of host organisms showed that globally, there is not a big difference in the fungal genes expressed (Baetsen-Young et al. 2020). Rather, some genes are upregulated much earlier in soybean compared to corn. These genes largely led to an inhibition of the host immune response to ensure successful root invasion. In corn, the genes are upregulated much later, probably due to poor host recognition, thereby giving corn more time to respond to the infection. Deletion of the transcription factor Pf2 in the plant pathogens *Alternaria brassicicola* and *Parastagonospora nodorum* and subsequent RNA-seq of infected roots showed that Pf2 regulates a network of fungal effector genes (e.g. plant cell wall-degrading enzymes) (Cho et al. 2013, Jones et al. 2019). The infection process of *B. cinerea* has been studied in dual RNA-seq experiments in *Fragaria vesca* and *A. thaliana*, where a race between plant immunity and (extracellular) fungal effector proteins was evident (Cho et al. 2013). Both studies show that fungi can suppress plant immunity early after infection before subsequently starting to release effectors that lead to plant cell death (Cho et al. 2013, Baetsen-Young et al. 2020).

In human fungal pathogens, a pioneering study investigated why organ/stem-cell transplant patients are at higher risk for invasive pulmonary aspergillosis after infection with human cytomegalovirus (CMV) (Garcia-Vidal et al. 2008) by establishing a triple RNA-seq approach (Seelbinder et al. 2020). *A. fumigatus* and CMV alone triggered quite different immune responses from monocyte-derived dendritic cells; however, during co-infection, fungal RNA expression was not influenced by the viral coinfection (or *vice versa*), but the dendritic cells exhibited a dampened immune response. This made the dendritic cells more susceptible to fungal infection, especially when CMV preceded the *A. fumigatus* infection.

The logical extension of such studies is to investigate the entire meta-transcriptome of a given microbial community, and in fact, such efforts are already underway. For example, simple variations have been performed under ideal laboratory conditions and more complex variations in the field (Marcelino et al. 2019, Auer et al. 2024). The next frontier will likely be spatial meta-transcriptomics to determine the abundance and localization of RNA molecules within complex communities. Although already reported, these exciting technologies are still being refined to improve sensitivity, reduce bias, and reach their full potential (Saarenpaa et al. 2024).

## Conclusions: where will technology lead us?

The frenetic pace of technological development in recent decades has transformed our ability to probe biological systems. Not only do we have a better understanding of these systems at the molecular level, but technology has facilitated development of exciting new drugs, e.g. the mRNA vaccines that carried us out of the pandemic. In thinking about the future of nucleic acids biology of fungal pathogens while writing this review, we were inspired by the questions at the precipice of our abilities. The possibilities are endless, but we would like to highlight a few areas where we hope for convergence of technology.

A major shift has occurred in the way we consider studying fungal pathogens, with a stronger emphasis placed on strain heterogeneity and studying pathogens in the context of the other species in their respective genera. Such comparisons have coincided with improved genomics capacity, enabling accumulation of many thousands of pathogen genomes and to a lesser extent, transcriptomes. The next step in this area appears to be centered on leveraging this treasure trove of sequence data, for example in the form of more complex pangenome assessments (Perrier and Barber 2024) or refined definitions of the transcriptome. Alongside, we expect more accurate descriptions of the difficult-to-assess areas of the genomes, like repetitive sequences encoded as parts of transposons or centromeres and telomeres. The few studies that have probed these areas are already revealing interesting permutations (Gervais and Shapiro 2024). Single-cell and spatial transcriptomic approaches are likely to further refine our understanding of population heterogeneity, both during host-pathogen interactions and in regard to environmental stress and antifungal drug tolerance.

We are also watching how nucleic acid-protein interaction technology progresses. Advanced LC-MS/MS-based proteomic methods are already being used to elucidate novel aspects of the proteome, from surface proteomes to intermolecular interactions (e.g. using BioID) or the post-translationally modified proteome (e.g. phospho-proteome). Many exciting questions remain, for example, how will improved sensitivity and high-throughput devices be leveraged to investigate the influence of post-translational protein modifications on nucleic acid biology? Another area seeing growth currently is in *ex vivo* models of fungal pathogens. The organ-on-chip systems, reviewed elsewhere (Alonso-Roman et al. 2024), provide sophisticated systems for investigation of microbial interactions with multiple host cellular partners. As these systems develop, we expect more complex analyses to be possible, for example, advanced sequencing approaches and RNA-localization or -trafficking studies. Coupled with cutting-edge microscopy platforms and improved imaging analysis workflows [e.g. JIPipe (Gerst et al. 2023)], we are likely to gain additional insight into the regulation of nucleic acids during infection. Large language models remain in their infancy, but efforts like Alphafold3 are already allowing for prediction of nucleic acid structures in association with binding proteins and will likely support data analysis of many other techniques described here. A future where the interactions between a protein and a DNA and/or RNA molecule (including modifications) can be predicted with high accuracy will be transformative. These artificial intelligence approaches are changing many aspects of the scientific process, and we can only imagine they will improve our ability to assess nucleic acids in fungi.

There are significant challenges ahead. For example, basic scientific research continues to be underfunded, even in countries where it is considered a priority. The biotech and pharmaceutical industries will continue to require improved technologies, but without investment in the discovery processes behind these innovations, it is hard to predict how the pace of development will change. Running parallel to these challenges is hopefulness. The promise of novel therapeutic strategies based on nucleic acids is clear, and improvements are rapidly appearing in the literature stemming from technological advances in RNA delivery methods (Li et al. 2025). The use of RNA-based medicines against human fungal pathogens is lagging, but in agriculture, the use of double-stranded RNA for spray-induced gene silencing has gained momentum in recent years to protect plants from fungal infections. Such tools will be important as we simultaneously deal with changing climate and emerging fungal pathogens in the coming decades.
